# The Onecut Transcription Factor HNF-6 Regulates in Motor Neurons the Formation of the Neuromuscular Junctions

**DOI:** 10.1371/journal.pone.0050509

**Published:** 2012-12-05

**Authors:** Emilie Audouard, Olivier Schakman, Frédérique René, Rosa-Eva Huettl, Andrea B. Huber, Jean-Philippe Loeffler, Philippe Gailly, Frédéric Clotman

**Affiliations:** 1 Institute of Neuroscience, Laboratory of Neural Differentiation, Université catholique de Louvain, Brussels, Belgium; 2 Institute of Neuroscience, Laboratory of Cell Physiology, Université catholique de Louvain, Brussels, Belgium; 3 INSERM U692, Laboratoire de Signalisations Moléculaires et Neurodégénérescence, Faculté de Médecine, Université de Strasbourg, Strasbourg, France; 4 Institute of Developmental Genetics, Helmholtz Zentrum München – German Research Center for Environmental Health, Neuherberg, Germany; Virginia Tech Carilion Research Institute, United States of America

## Abstract

The neuromuscular junctions are the specialized synapses whereby spinal motor neurons control the contraction of skeletal muscles. The formation of the neuromuscular junctions is controlled by a complex interplay of multiple mechanisms coordinately activated in motor nerve terminals and in their target myotubes. However, the transcriptional regulators that control in motor neurons the genetic programs involved in neuromuscular junction development remain unknown. Here, we provide evidence that the Onecut transcription factor HNF-6 regulates in motor neurons the formation of the neuromuscular junctions. Indeed, adult *Hnf6* mutant mice exhibit hindlimb muscle weakness and abnormal locomotion. This results from defects of hindlimb neuromuscular junctions characterized by an abnormal morphology and defective localization of the synaptic vesicle protein synaptophysin at the motor nerve terminals. These defects are consequences of altered and delayed formation of the neuromuscular junctions in newborn mutant animals. Furthermore, we show that the expression level of numerous regulators of neuromuscular junction formation, namely *agrin*, *neuregulin-2* and *TGF-ß receptor II*, is downregulated in the spinal motor neurons of *Hnf6* mutant newborn animals. Finally, altered formation of neuromuscular junction-like structures in a co-culture model of wildtype myotubes with mutant embryonic spinal cord slices is rescued by recombinant agrin and neuregulin, indicating that depletion in these factors contributes to defective neuromuscular junction development in the absence of HNF-6. Thus, HNF-6 controls in spinal motor neurons a genetic program that coordinates the formation of hindlimb neuromuscular junctions.

## Introduction

The neuromuscular junctions (NMJ) are the synapses whereby spinal motor neurons (MN) control the contraction of skeletal muscles. The development of the NMJ is initiated by interaction of the growing terminal ends of MN, guided by pre-existing acetylcholine receptor (AChR) clusters at the muscle surface, with the plasma membrane of the myotubes. This initial interaction favors local clustering of AChR and stimulates in the myotubes the expression of genes encoding post-synaptic components. Organization of these components into a post-synaptic apparatus results in a concomitant remodeling of the motor terminal ends that ensures perfect apposition of pre-synaptic and post-synaptic complements [1], [2], [3]. Terminal Schwann cells surrounding the NMJ also contribute to support their formation and maintenance [4]. In limb muscles, NMJ formation starts around embryonic day (e) 16.5 and is completed around postnatal day (P) 14.

NMJ formation is controlled by a complex interplay of multiple mechanisms coordinately activated in motor nerve terminals and in their target myotubes [3]. Agrin, a heparan sulfate proteoglycan, is released from nerve terminals and, through interaction with the low-density lipoprotein receptor-related protein 4, activates the Muscle-Specific Kinase complex. This stimulates the organization of the postsynaptic apparatus and, through rapsyn, clustering of the AChR. Hence, agrin counteracts the dispersal of AChR induced by ACh, contributing to stabilize the NMJ [5]. Neuregulins also contribute to stabilize the post-synaptic apparatus [6] and are required for development of the terminal Schwann cells that in turn promote NMJ maintenance [7], [8]. Indeed, recent data suggest that terminal Schwann cells secrete TGF-ß that could increase agrin expression in MN [9]. Additional MN-secreted factors including members of the Wnt family and muscle derived signals like FGFs, GDNF and extracellular matrix proteins also contribute to NMJ maturation, stabilization and maintenance [10], [11], [12].

In MN, the transcriptional regulators that control the genetic programs involved in NMJ formation or maintenance remain unknown. Here, we provide evidence that the transcription factor Hepatocyte Nuclear Factor-6 (HNF-6) acts in MN to regulate the formation of the NMJ. HNF-6 belongs to the Onecut family of transcriptional activators [13], [14], [15], [16]. These factors control cell differentiation and tissue morphogenesis in developing liver and pancreas [17], [18], [19], [20], [21], [22]. Furthermore, they regulate neuronal identity, migration, maintenance or projections in different regions of the CNS [23], [24], [25], [26], [27] Onecut factors are present in MN from the onset of their differentiation until birth [28].

Here, we present evidence that HNF-6 acts in MN to regulate the formation of the NMJ. Indeed, mouse mutants for *Hnf6* exhibit hindlimb locomotor defects associated with abnormal morphology of the NMJ and defective localization of the synaptic vesicle protein synaptophysin. These result from defective development and maturation delay of the hindlimb NMJ in the early post-natal period. Furthermore, the expression of factors essential for the formation or maturation of the NMJ is downregulated in *Hnf6−/−* lumbar MN, and these perturbations contribute to the mutant phenotype. Thus, HNF-6 controls in MN a genetic program that coordinates the formation of hindlimb neuromuscular junctions.

## Materials and Methods

### Animals

All experiments were strictly performed respecting the European Community Council directive of 24 November 1986 (86-609/ECC) and the decree of 20 October 1987 (87-848/EEC). All mice were raised on our animal facilities and were treated according to the principles of laboratory animal care of the Animal Welfare Committee of the Université catholique de Louvain (Permit Number: UCL/MD/2009/008). Electromyography recordings, tissue perfusions and cervical dislocation were performed under ketamine/xylazine anesthesia, and all efforts were made to minimize suffering. *Hnf6* knockout (*Hnf6−/−*) mice were obtained as described [19]. As *Hnf6+/−* mice do not exhibit any abnormality, control animals will refer to either wildtype or *Hnf6+/−* individuals. Mice were genotyped by PCR (primers available upon request).

### Behavioral Tests

**Figure 1:**
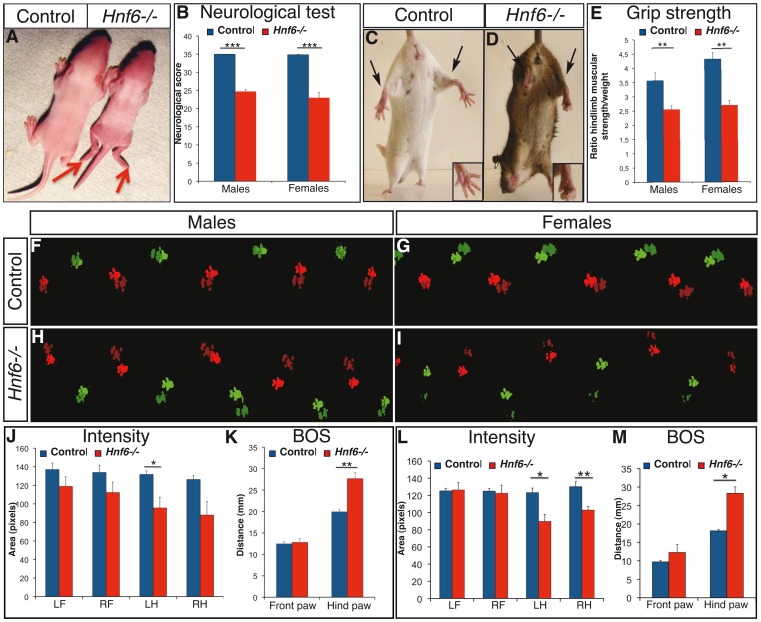
Absence of HNF-6 results in abnormal locomotion. ***A***, Newborn *Hnf6−/−* mice (right) are smaller than their control littermates (left) and show paresis of the hindlimbs (arrows). ***B***, Neurological score of male or female control (blue) or *Hnf6−/−* (red) mice. The score is reduced in both male and female *Hnf6−/−* mice (n≥7). ***C***, ***D***, *Hnf6−/−* mice (D) exhibit a feet-clasping posture of the hindlimbs upon tail suspension, both for the legs (arrows) and for the feet (inset). ***E***, The hindlimb muscular strength of male and of female control (blue) or *Hnf6−/−* (red) mice normalized to the body weight is reduced in *Hnf6−/−* mice (n≥4). ***F–M*** **,** Catwalk analyses were performed for male (F,H,J,K) or female (G,I,L,M) control (F,G,J–M) or *Hnf6−/−* (H,I,J–M) mice. Locomotion alterations characterized by reduced overlap of fore paw (light colors) and of hind paw (dark colors) prints, reduced area of hind paw contacts, variability in the distance between the left (green) and the right (red) prints and less linear displacement, are observed in *Hnf6−/−* mice. Quantifications indicate that the intensity of the hind paw contacts is reduced (J,L) while the hindlimb base of support (the distance between the left and right hind paws) is increased (K,M). In contrast, these parameters are not affected for the forelimbs (n≥4 in each group). BOS: base of support; LF: left front; LH: left hind; RF: right front; RH: right hind. Student’s t-test; * = p<0.05; ** = p<0.01; *** = p<0.001.

All mice were handled and trained for at least two weeks before starting experiments. Animals were weighted each experiment day. Behavioral tests were applied separately for male and for female control or *Hnf6−/−* mice, starting when animals were aged 2 months. They were performed during five weeks. At birth, the *Hnf6−/−* mice were smaller than control littermates (Fig. 1A) and showed a reduced growth rate [19]. At later stages, this difference progressively normalized and around three months, the average weight of *Hnf6−/−* mice was similar to that of control mice (Fig. S2M).

The global neurological status of the animals was estimated based on a neurological score [29]. This neuroscore comprises 11 parameters with a maximal cumulative score of 35: general condition (maximum 4 points), paw placement onto a table top (maximum 4 points), ability to pull itself up on a horizontal bar (maximum 3 points), turnaround reflex (maximum 2 points), climbing on a inclined platform (maximum 4 points), grasp reflex (maximum 4 points), contralateral rotation when held by the base of tail (maximum 1 point), paw positioning (maximum 8 points), tail positioning (maximum 1 point), tilt head (maximum 1 point) and circling (maximum 3 points). For each parameter, the score was determined on a scale starting from 0 for severe impairment to the maximum score for healthy function. This score was applied to control or *Hnf6−/−* mice aged 3 months.

The force of the hindlimb muscles was measured using an animal grip strength system (San Diego Instruments, USA), as described [30]. The Catwalk test was used to assess the gait of voluntarily walking mice (Catwalk 7, Noldus; Wageningen, The Netherlands) [31], [32]. Five runs were performed per animal and gait parameters were taken into account only when the run time was inferior to 10 s [33].

### Tissue Preparation

Mice were anesthetized with ketamine (Ketalar 75 mg/kg) and xylazine (Rompun 5 mg/kg) solution. Mice were perfused transcardially with 4% paraformaldehyde (PFA) in phosphate-buffered saline (PBS). Limbs muscles and the lumbar portion of the spinal cord were dissected, the latter was post-fixed overnight at 4°C in 4% PFA/PBS. Embryos at e18.5 were fixed in 4%PFA/PBS/0.002%NP40 at 4°C for 1 hour. Samples were rinsed three times in PBS and cryoprotected in 30% sucrose/PBS, except for limbs muscles in 20% sucrose/PBS, for 48 hours. Tissues were embedded in Tissue Tek (Prolabo, VWR) and cut (20 µm) using a Microm HM500 cryostat. Cryosections were dried at room temperature and stored at −20°C.

### Histological and Immunofluorescence Labelings, and Quantifications

Tibialis anterior (TA), soleus, gastrocnemius and extensor digitorum longus (EDL) muscles were dissected at P70 and weighted. They were formalin-fixed, paraffin-embedded, cut (10 µm) and stained with hematoxylin/eosin. Pictures were acquired with an Olympus BX 60 fluorescence microscope (Camera Olympus XM10) with CellˆF software. The muscle fiber cross-sectional area (CSA) was measured on 200 fibers for TA and 100 fibers for soleus in each animal (n = 3) using the ImageJ software. Immunofluorescence labelings on spinal cord cryosections were performed as described [28]. Muscle cryosections were treated with 0.1 M glycine in PBS for 30 min before processing. After washes in PBS, sections were permeabilized with PBS/0.3% TritonX-100 for 10 min and saturated with PBS/0.3% Triton/10% BSA for 45 min. The primary antibodies were diluted in the saturation solution and incubated O/N at 4°C. After washes in PBS/0.1% Triton, the secondary antibodies and α-bungarotoxin were diluted in PBS/0.1% Triton/10% BSA and added for 1 hour at room temperature. After washes in PBS/0.1% Triton, the slides were mounted with fluorescent mounting medium (DAKO, Via Real, Capinteria, CA, USA). Diaphragms were stained whole-mount as previously described [34]. Primary antibodies for immunofluorescence were the mouse anti Pan-neurofilament (1∶2000; Covance, SMI-312), guinea pig anti vesicular acetylcholine transporter (VAChT; 1∶500; Millipore, AB1588), mouse anti synaptic vesicle (SV2, 1∶500; DSHB), rabbit anti synaptophysin (1∶250, Life Technologies, #18013), guinea pig anti hepatocyte nuclear factor-6 (HNF-6; 1∶2000; [24]). Motor neurons were labeled with mouse anti Isl1/2 (1∶6000, DSHB, #39.3F.7), goat anti VAChT (1∶500, Millipore, AB1578) or rabbit anti Hb9 (1∶1000, Abcam, ab26128). Fluorogold (1%; Fluorochrome, Denver, CO) was injected i.p. 5 days before perfusion [35]. Secondary antibodies were diluted 1∶2000 and were donkey anti guinea pig/AlexaFluor 488, 594 or 647, anti mouse/AlexaFluor 488, 594 or 647, anti goat/AlexaFluor 488 and anti rabbit/AlexaFluor 488 or 594 (Life Technologies, Carlsbad, CA, USA). Alpha-bungarotoxin-Alexa594 (1∶2000; Life Technologies, B13422) was incubated with the secondary antibodies. Pictures were taken with a Zeiss Cell Observer Spinning Disk confocal microscope (Carl Zeiss, Zaventem, Belgium). For all images, brightness and contrast were adjusted with Adobe Photoshop CS3 extended version 10.0 after acquisition to match with the observation.

MN were quantified on each side of 3 spinal cord sections stained with cresyl violet or for Hb9 or Isl1/2 from a minimum of 3 mice. NMJ morphology was quantified on a minimum of 100 hindlimb junctions from 3 different animals. Synaptophysin distribution was classified into four categories: 1) perfect superposition between the BTX and synaptophysin labelings, 2) fragmentation of synaptophysin labeling compared to BTX staining, 3) weak synaptophysin labeling and 4) absence of synaptophysin. A minimum of 400 junctions was analyzed for each animal (n = 6). Motor endplate maturation was evaluated in P14 newborn mice based on criteria previously described [36], [37]. A minimum of 100 junctions was analyzed for each animal (n = 3).

### Electronic Microscopy

Mice were anesthetized as mentioned above and perfused with a 2% PFA- 2.5% glutaraldehyde- 0.1 M phosphate buffer- 7% saccharose- 4% polyvinylpyrolidone solution. TA muscles were dissected and were conserved in the perfusion solution at 4°C. Fixed tissues were then rinsed in 0.1 M phosphate buffer and postfixed for 1h30 with 1% osmium tetroxide in the same buffer. They were dehydrated in ethanol (30% to 100%) and embedded in Epon. Longitudinal thin sections (70 nm) were obtained with a LEICA Ultracut T and collected on 150-mesh copper grids, double stained with uranyl acetate and lead citrate, before examination with electron microscope HITACHI H7650 TEM at 80 kV. Images were collected with an Orius camera with GATAN Digital Micrograph software.

### Electromyography

Electromyography recordings were made using a portable Dantec system (Dantec, Les Ulis, France) and were performed as described [38]. Briefly, mice were anesthetized as mentioned above and electrical activity in gastrocnemius and in TA muscles was monitored.

### RT-qPCR

Total RNA was extracted from P0 hindlimb muscles, liver or spinal cord using the Tripure Isolation Reagent (Roche). One microgram of total RNA was transcribed into cDNA with the First-Strand Synthesized System (Life Technologies) according to the manufacturers instructions. cDNA was amplified with Platinum SYBR Green qPCR SuperMix-UDG (Life Technologies). Primers for RT-qPCR were: agrin isoform Z, which is the isoform present in neurons (forward 5′-GACGCAGTGGTTCCTTCTGT-3′, reverse 5′-ACGAGCCAAGAACACCATCT-3′), neuregulin-1 (forward 5′-CAGGAACTCAGCCACAACA-3′, reverse 5′-TGACAGGTCCTTCACCATGA-3′), neuregulin-2 (forward 5′-ATCATCATCTCCGGCAGAAC-3′, reverse 5′-GAGGTCAGGCTCTCTGAACG-3′), TGF-ß Receptor II [39], choline acetyltransferase (forward 5′-CCGGTTTATTCTCTCCACCA-3′, reverse 5′-GGTTGGGCCTCTAGCTCTTT-3′), hepatocyte nuclear factor-6 (forward 5′-TTCCAGCGCATGTCGGCGCTC-3′, reverse 5′-GGTACTAGTCCGTGGTTCTTC-5′) and RPL 32 (forward 5′-GGCACCAGTCAGACCGATAT-3′, reverse 5′-CAGGATCTGGCCCTTGAAC-3′) as internal standard. The amplification protocol for all the primers included a hot start (95°C for 8 min 30 s), 40 amplification cycles (95°C for 15 s, 58°C [*Agrin*, *Neureugulin-2*, *Choline acetyltransferase*, *RPL 32*] or 60°C [*TGF-ß Receptor II*, *Neuregulin-1* and *Hepatocyte nuclear factor-6*] for 1 min) and a melt-curve analysis. Data were analyzed using the iCycler software with the delta/delta CT method and normalized to RPL32.

### *In situ* Hybridization

*Choline acetyltransferase* and *Vesicular Acetylcholine Transferase* riboprobes were provided by Dr. Ernsberger [40], *Neuregulin-1* and *Neuregulin-2* by Dr. C. Lai [41] and *TGF-ß Receptor II* by Dr. F. Lemaigre [42]. The *Agrin* riboprobe corresponded to nucleotides 6327–6840 of the NM_021604.3 NCBI sequence. *In situ* hybridizations on sections were performed as described [43] and followed by immunofluorescence detection of Isl1/2 as described above. Pictures were taken with an AMG Evosfl digital inverted microscope.

### Co-culture of Skeletal Muscle Cells and Embryonic Spinal Cord Slices

Hindlimb muscles from P0–P5 animals were dissected, minced and incubated at 37°C for 30 min in 0.0175% collagenase (Life Technologies), followed by 2 incubations in 0.05% trypsin-EDTA (Life Technologies) at 37°C for 15 min. Supernatants were filtered. The dissociated cells were resuspended in growth medium (DMEM-F12, 30% foetal bovine serum, 2 mM glutamine, 1% penicillin-streptomycin [Life Technologies]). Cells were plated onto 12-well plates coated with 2% gelatin (Sigma) and cultured in a humidified atmosphere at 37°C in 5% CO_2_. Myoblast differentiation and fusion was induced in differentiation medium (DMEM, 2 mM glutamine, 1% penicillin-streptomycin, 10% horse serum and 10 µg/ml insulin [Sigma]) for 7–9 days.

Mouse embryos collected at e12.5 were embedded into low-melting point agarose (Ultrapure LMP agarose, Life Technologies) and 300 µm-thick sections were prepared using a NVSLM1 vibroslice vibratome (World Precision Instruments). Spinal cord slices with attached dorsal root ganglia were isolated and plated onto the myotube cultures. Two to three spinal cord sections were placed into each well and medium was replaced by DMEM, 5% foetal bovine serum, 2 mM L-glutamine, 1% penicillin-streptomycin for 2–3 weeks supplemented with recombinant rat Agrin (R&D systems, #550-AG, 10 ng/ml), recombinant human NRG1-ß1/HRG1-ß1 extracellular domain which activates the same receptors as Nrg2 (R&D systems, #377-HB, 10 ng/ml) or recombinant human TGF-ß1 (R&D systems, #240-B, 100 pg/ml) for rescue experiments.

Co-cultures were fixed with 4% PFA/PBS at room temperature for 30 min, then washed three times in PBS. They were saturated with PBS/1% BSA/0.1% Triton for 30 min and washed with 1%BSA/PBS. The α-bungarotoxin-Alexa594 (1∶2000; Life Technologies) was incubated in PBS/1% BSA solution for 1 hour at room temperature and rinsed in PBS. Pictures were taken with an AMG Evos fluorescence microscope. The length and the surface of the NMJ-like structures were measured using the ImageJ software.

### Statistics

Data are presented as mean values ± SEM. Unpaired Student’s t-test, χ^2^ Pearson’s test, Dunett’s or Newman-Keuls multiple comparison tests were used to determine statistical significance using GraphPad Prism 4 software (San Diego, CA). Differences at p<0.05 were considered significant.

## Results

### Absence of HNF-6 Results in Abnormal Locomotion

At birth, mice carrying a null mutation of *Hnf6* exhibited a paresis of the hindlimbs (Fig. 1A). To determine the outcomes of this early abnormality, a neurological score based on 11 criteria (see Material & Methods) that enabled a global assessment of the neurological status of these mice was evaluated. Male and female *Hnf6−/−* mice aged 70 days showed a significant reduction in this score compared to control littermates (Fig. 1B). In particular, feet-clasping posture of the hindlimbs upon tail suspension was observed for mutant mice (Fig. 1C,D), suggestive of hindlimb muscle weakness. Therefore, limb muscular strength was evaluated using grip strength measurements. A strong reduction in hindlimb muscular strength, but not in forelimb strength (data not shown), was observed in male and in female *Hnf6−/−* mice. To ascertain that the observed strength reduction was not due to size differences, grip strength measurements were normalized for body weight. Nevertheless, the normalized values showed a 30 or 40% reduction in hindlimb muscular strength for *Hnf6−/−* male or female mice, respectively (Fig. 1E).

To determine whether this muscle weakness altered the locomotion of the mutant mice, gait parameters were evaluated by catwalk imaging. Compared to control mice, mutant individuals exhibited little overlap of fore paw and hind paw prints, reduced area of hind paw contacts, variability in the distance between the left and the right prints and less linear displacement (Fig. 1F–I). Quantitative analyses of gait parameters confirmed a reduction in the intensity of hind paw contacts and an increase in the base of support (BOS, i.e. the distance between the left and right paws) of the hindlimbs, both for males and for females (Fig. 1J–M), indicative of hindlimb muscle weakness and posture defect. Other hindlimb parameters were normal, and forelimb parameters were not affected. Taken together, these observations suggest that the lack of HNF-6 causes locomotor alterations that result from muscle weakness and abnormal positioning of the hindlimbs.

### Absence of HNF-6 Results in Muscle Hypotrophy or Hypertrophy

**Figure 2:**
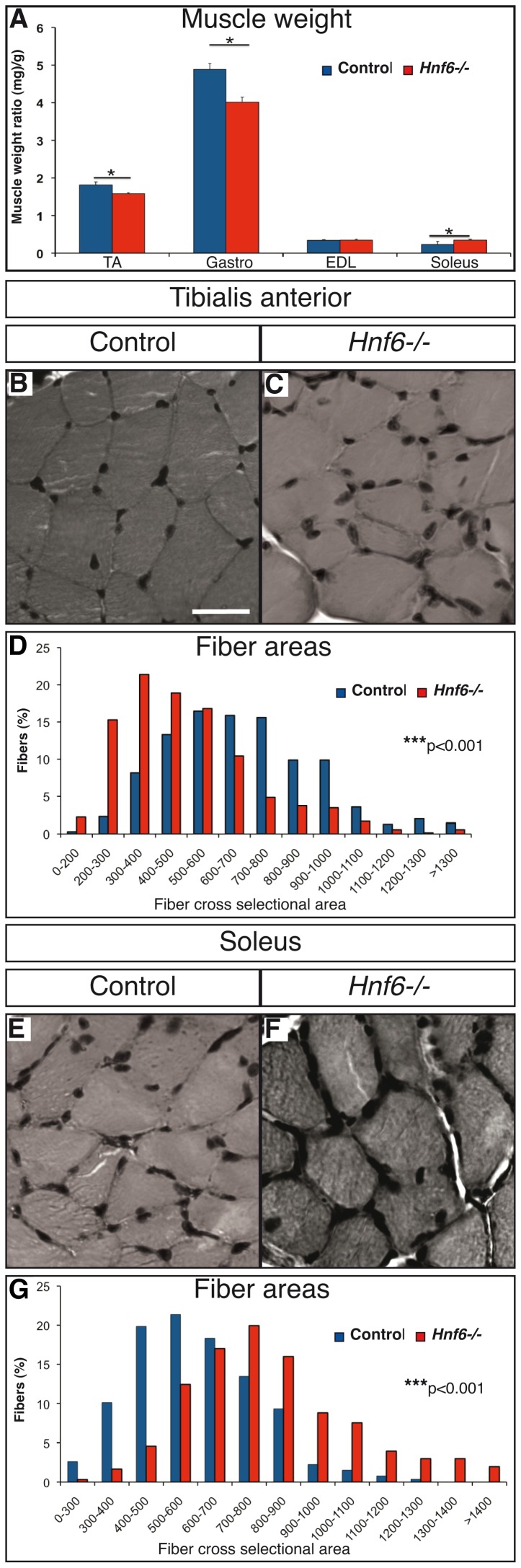
Absence of HNF-6 results in muscle hypotrophy or hypertrophy. ***A***, Tibialis anterior, gastrocnemius, extensor digitorium longus and soleus muscles from control (blue) or *Hnf6−/−* (red) mice were weighted. The tibialis anterior and the gastrocnemius are hypotrophied in *Hnf6−/−* mice, whereas the soleus is hypertrophied. ***B*** *,* ***C***, Hematoxylin-eosin stainings of transverse sections in tibialis anterior from control (B) or *Hnf6−/−* (C) mice does not evidence any sign of muscle degeneration/regeneration but shows a reduction in fiber size. ***D***, Distribution of fiber area in the tibialis anterior of control (blue) or *Hnf6−/−* (red) mice. The tibialis anterior fibers are smaller in the absence of HNF-6. ***E*** *,* ***F***, Hematoxylin-eosin staining of transverse sections in the soleus of control (E) or *Hnf6−/−* (F) mice shows an increase in fiber size. ***G*** **,** Distribution of fiber area in the soleus of control (blue) or *Hn6−/−* (red) mice. The soleus fibers are bigger in the absence of HNF-6. Gastro: gastrocnemius; EDL: extensor digitorium longus; TA: tibialis anterior. (n = 3) Student’s t-test (A), χ^2^ Pearson’s test (D,G), * = p<0.05; *** = p<0.001. Scale bar = 50 µm.

Hindlimb muscle weakness in *Hnf6−/−* animals may result from muscle degeneration and wasting. To address this question, four hindlimb muscles were weighted and the muscle fiber cross-sectional area (CSA) was measured. The TA and gastrocnemius were smaller in *Hnf6−/−* individuals. In contrast, the soleus was slightly bigger, whereas EDL weight was normal (Fig. 2A). In *Hnf6−/−* animals, the mean CSA in TA was reduced without modification in the number of fibers (Fig. 2B–D) whereas CSA in soleus was increased without change in fiber number (Fig. 2E–G). Absence of central nuclei suggested that *Hnf6−/−* muscles did not undergo degeneration/regeneration (Fig. 2C,F). Taken together, these data indicated that the absence of HNF-6 resulted in hypertrophy of the soleus and in hypotrophy of the TA and of the gastrocnemius. Marked hypotrophy of these two large muscles likely contributes to the observed hindlimb muscle weakness.

### HNF-6 is Required for Proper Morphology of the NMJ

**Figure 3:**
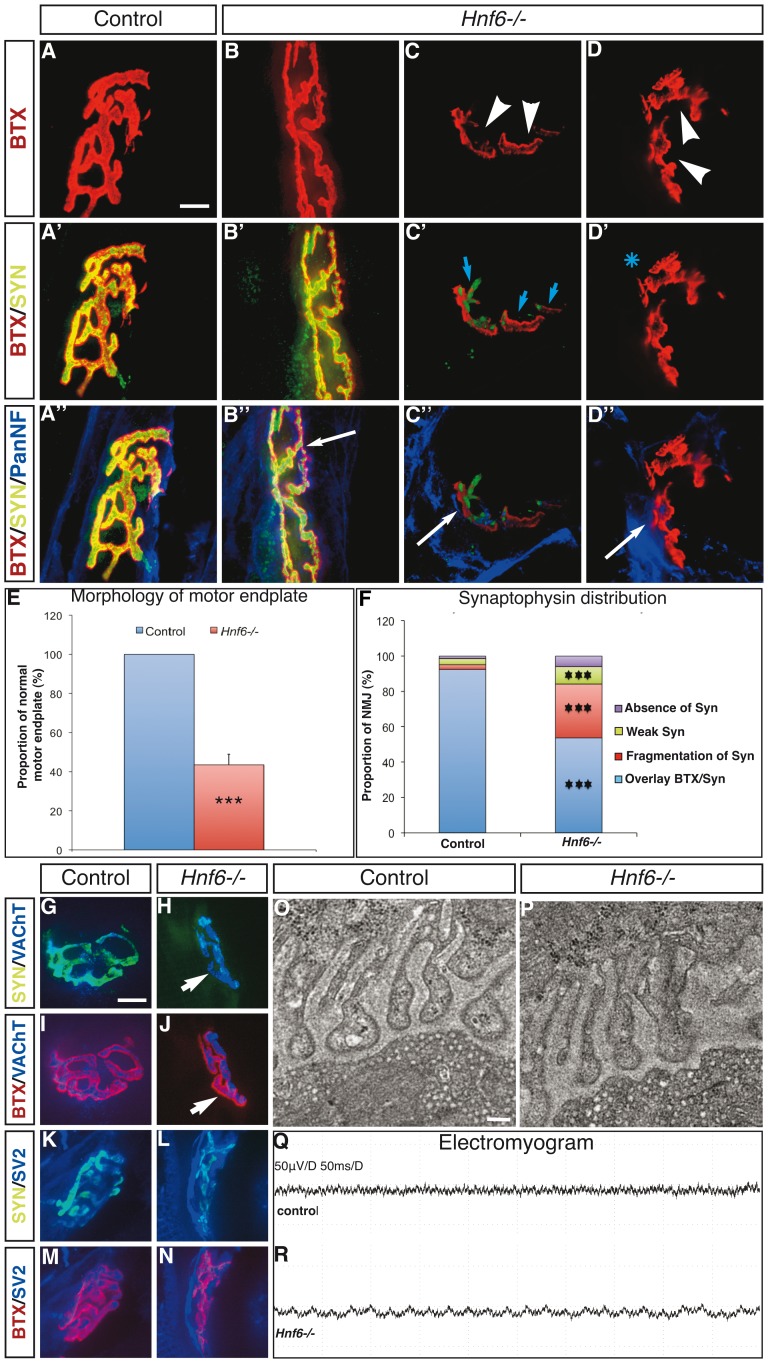
HNF-6 is required for proper morphology of the NMJ. ***A–D”*** **,** Labeling of acetylcholine receptors by α-bungarotoxin (red) and immunofluorescence detection of synaptophysin (green) and of neurofilaments (blue) on hindlimb sections of control (A–A”) or *Hnf6−/−* (B–D”) mice. (A–A”) In control mice, NMJ display the expected “pretzel-like” shape, are innervated and show perfect apposition of the synaptophysin labeling to the motor endplate. (B–B”) Some NMJ in *Hnf6−/−* mice are very similar to that observed in control animals. (C–D”) However, a majority of the *Hnf6−/−* junctions are characterized by disorganized topology, fragmentation or absence of gutters (arrowheads). These junctions also show defective localization of synaptophysin, which is either absent (asterisk) or fragmented (blue arrows) and ectopically located. In contrast, all the junctions were innervated (white arrows). ***E*** **,** Quantification of motor endplates that exhibit the expected “pretzel-like” unfragmented morphology in control (blue) and in *Hnf6−/−* (red) mice (n = 6). **F,** Quantification of defective synaptophysin localization in *Hnf6−/−* mice. Synaptophysin is either properly superposed to the α-bungarotoxin labeling, as observed in a vast majority of control junctions (blue), or fragmented (red), weak (green) or absent (purple) (n = 5). ***G–N*** **,** Labeling of control (G,I,K,M) or *Hnf6−/−* (H,J,L,N) junctions with synaptophysin (G,H,K,L, green) or α-bungarotoxin (I,J,M,N, red) and VAChT (G–J, blue) or SV2 (K–N, blue). In *Hnf6−/−* mice, the VAChT and SV2 are properly localized at the motor terminal ends. ***O, P*** **,** Ultrastructure of the NMJ observed by transmission electron microscopy in control (O) or *Hnf6−/−* (P) tibialis anterior muscles. Synaptic vesicles are present and normally localized (n = 3). ***Q, R*** **,** Electromyogram recordings in control (Q) or *Hnf6−/−* (R) mice do not evidence any sign of denervation of the mutant hindlimb muscles (n = 3). BTX: α-bungarotoxin; PanNF: Pan-Neurofilament; SV2: synaptic vesicle 2; SYN: synaptophysine; VAChT: vesicular acetylcholine transporter. Student’s t-test; *** = p<0.001. Scale bar in A and G = 5 µm; scale bar in O = 0.4 µm.

However, HNF-6 is not detectable in muscles [44], either by RT-PCR or by immunofluorescence (Fig. S1). This also indicates that it is not present in cells that engulf the NMJ, including terminal Schwann cells [45] and kranocytes [46]. In contrast, HNF-6 is detected in MN at each level along the antero-posterior axis of the spinal cord during development [28] and in the early post-natal period (Fig. S1). In the limb-innervating lateral motor columns, HNF-6 is restricted to subsets of MN that do not correspond to motor pools, which innervate specific limb muscle [28]. This suggested that MN defects may constitute the primary cause of the locomotion defects in *Hnf6−/−* mice. The amount, the molecular phenotype and the projections of lumbar MN were preserved in *Hnf6−/−* mice (Fig. S2). Therefore, we investigated the morphology of the NMJ in all the hindlimb muscles. Labeling of the AChR by α-bungarotoxin (BTX) unveiled that 56% of the NMJ exhibited morphological alterations in *Hnf6−/−* mice (Fig. 3A–E) including disorganized topology (Fig. 3C,D), fragmentation (Fig. 3C) or absence of gutters (Fig. 3D). These abnormalities were observed in the thigh, TA, EDL and gastrocnemius muscles (Fig. S3). In contrast, all NMJ were normal in hindlimb soleus (Fig. S3), in forelimb muscles and in the diaphragm (Fig. S4). This indicated that HNF-6 is required for proper organization of a large proportion of the hindlimb NMJ, and suggested that NMJ alterations likely participate in the locomotion defects of *Hnf6−/−* mice.

### HNF-6 is Required for Proper Localization of Synaptophysin at Motor Nerve Terminals

To further investigate the organization of the NMJ in the absence of HNF-6, sections in all the hindlimb muscles were additionally labeled for the MN presynaptic vesicular marker synaptophysin, and for neurofilaments to stain the motor axons. All the NMJ of *Hnf6−/−* mice were innervated (Fig. 3B”–D”), consistent with proper innervation of the limb anlagen observed during embryonic development [27]. However, the localization of synaptophysin was defective in the absence of HNF-6. In control muscles, 92±2% of the NMJ showed extensive superposition of BTX and of synaptophysin labelings (Fig. 3A’,F), indicative of precise apposition of motor nerve terminals to the motor endplates. In contrast, only 54±5% of the NMJ exhibited precise superposition of BTX and of synaptophysin labelings in *Hnf6−/−* mice (Fig. 3B’,F). In the other cases, synaptophysin was either fragmented, that is, only partly superposed to the BTX labeling (Fig. 3C’,F), detected at very low levels (Fig. 3F) or absent (Fig. 3D’,F). Furthermore, synaptophysin was frequently ectopically located, i.e. adjacent but not superposed to the BTX labeling (Fig. 3C”). Occasionally, it also colocalized with neurofilaments inside the terminal portion of the MN axons (Fig. 3B”). Although widely associated with NMJ morphological alterations, defective synaptophysin localization was also observed at morphologically normal NMJ (Fig. 3B”,L).

These observations suggested that the absence of HNF-6 resulted in defective apposition of the motor nerve terminals to the motor endplates, perturbed addressing of the presynaptic vesicles to the motor terminals or abnormal distribution of synaptophysin. To address these possibilities, muscle sections were labeled for other presynaptic vesicle proteins including VAChT and SV2. However, VAChT and SV2 labelings were precisely superposed to BTX staining in control and in *Hnf6−/−* hindlimb muscles (Fig. 3G–N). This suggested that the motor nerve terminals are superposed to motor endplates and that presynaptic vesicles are correctly localized at the terminal ends of MN in the absence of HNF-6. To confirm this, the ultrastructure of the NMJ in *Hnf6−/−* TA muscles was analyzed by transmission electron microscopy. Consistent with normal distribution of VAChT and SV2, synaptic vesicles were present and properly localized beneath the presynaptic membrane in all the junctions of TA *Hnf6−/−* samples (Fig. 3O,P). These data indicated that, despite alterations of morphology and of synaptophysin distribution, hindlimb NMJ in *Hnf6−/−* mice might be functional. To address this hypothesis, electromyographic recordings of the gastrocnemius and of the TA were performed in control and in mutant mice. Consistent with the electron microscopy data and with normal innervation of the NMJ, electrical activity recordings of *Hnf6−/−* muscles did not show any sign of denervation (Fig. 3Q,R). These observations confirmed that NMJ were properly innervated and likely functional in *Hnf6−/−* mice. Thus, HNF-6 is required for proper morphology and for correct distribution of synaptophysin at motor nerve terminals in about half of the hindlimb NMJ.

### HNF-6 is Required for Timely Formation and Maturation of the NMJ

**Figure 4:**
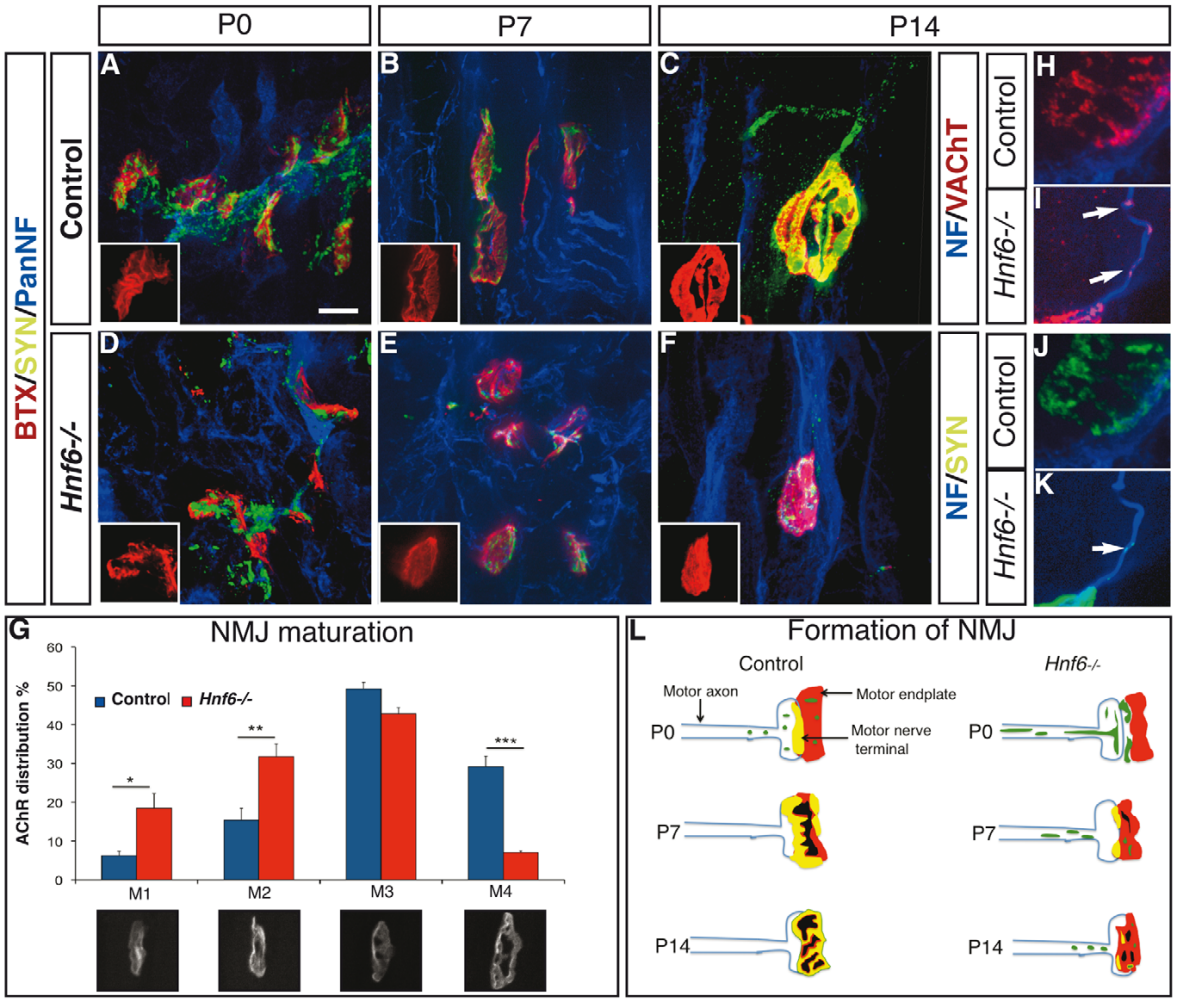
HNF-6 is required for the formation of NMJ. ***A–F*** **,** Labeling of acetylcholine receptors by α-bungarotoxin (red) and immunofluorescence detection of synaptophysin (green) and of neurofilaments (blue) on hindlimb sections of control (A–C) or *Hnf6−/−* (D–F) mice at postnatal day 0 (A,D), 7 (B,E) or 14 (C,F). The progressive increase in apposition of the motor nerve terminals to the motor endplates is disrupted in the absence of HNF-6, and the maturation of the motor endplate is defective. ***G*** **,** Quantification of NMJ maturation in control (blue) or *Hnf6−/−* (red) mice based on 4 morphological stages (M1: oval plaque; M2: single perforation; M3: multiple perforations; M4: mature junction). The maturation of the junctions is delayed in the absence of HNF-6 (n = 3). ***H–K*** **,** Labeling of VAChT (H,I, red) or synaptophysin (J,K, green) and of neurofilaments (blue) in control (H,J) or *Hnf6−/−* (I,K) mice at postnatal day 14. VAChT and synaptophysin were present in the terminal portion of motor axons in *Hnf6−/−* mice (I,K) whereas they were restricted to nerve terminals in control mice (H,J). ***L*** **,** Schematic representation of NMJ formation in control and in *Hnf6−/−* mice. BTX: α-bungarotoxin; PanNF: Pan-neurofilament; P: postnatal day; SYN: synaptophysin; VAChT: vesicular acetylcholine transporter. Student’s t-test, * = p<0.05, ** = p<0.01, *** = p<0.001. Scale bar = 5 µm.

Half of the hindlimb NMJ were abnormal in adult *Hnf6−/−* mice. To assess whether this was due to defective formation or to abnormal stabilization and maintenance of the NMJ, the development of the NMJ in *Hnf6−/−* hindlimb muscles was studied between birth and P14. In control newborns at P0, AChR were arranged in plaques overlaid with sparse dotted synaptophysin aggregates likely corresponding to presynaptic vesicles (Fig. 4A). At P7, motor endplates reorganized and started to display perforations while matching between motor nerve terminals and endplates increased (Fig. 4B). At P14, the most mature junctions displayed a “pretzel-like” shape while synaptophysin was superposed to the endplate gutters, indicative of a precise apposition of the nerve terminals to the motor endplates (Fig. 4C). In contrast, the formation of the NMJ in hindlimb muscles was severely affected and delayed in *Hnf6−/−* newborns. At P0, AChR were normally organized in plaques but synaptophysin was clustered and was not superposed to the motor endplates (Fig. 4D). At P7, motor endplates presented very few perforations, with sparse superposition of the synaptophysin labeling (Fig. 4E). At P14, a majority of NMJ displayed a round morphology lacking any “pretzel-like” organization, while synaptophysin labeling was fragmented (Fig. 4F). These observations indicated that, in the absence of HNF-6, synaptophysin does not properly localize at the motor nerve terminals and that the maturation of the NMJ seems to be delayed. To confirm this, maturation of the NMJ was evaluated at P14 based on four morphological stages [47]. This quantification showed an excessive amount of NMJ in an immature configuration (M1–M2) and fewer mature NMJ in *Hnf6−/−* mice (Fig. 4G), confirming the maturation delay of the junctions in these animals. Maturation delay of the NMJ was previously associated with abnormal clustering of presynaptic vesicle proteins, including VAChT and synaptophysin, in the terminal MN axonal compartment [10]. Accordingly, synaptophysin and VAChT were ectopically detected within MN axons at P14 in *Hnf6−/−* newborns, whereas they were excluded from the terminal axonal compartment in control animals (Fig. 4H–K). Taken together, these observations suggested that NMJ formation is altered and delayed in hindlimb muscles of *Hnf6* mutant mice (Fig. 4L). Given that a large proportion of the hindlimb NMJ was abnormal in *Hnf6−/−* adult mice, it is likely that these early defects hamper final maturation of the junctions.

### HNF-6 Regulates in Motor Neurons the Expression of Several Actors Involved in NMJ Formation

**Figure 5:**
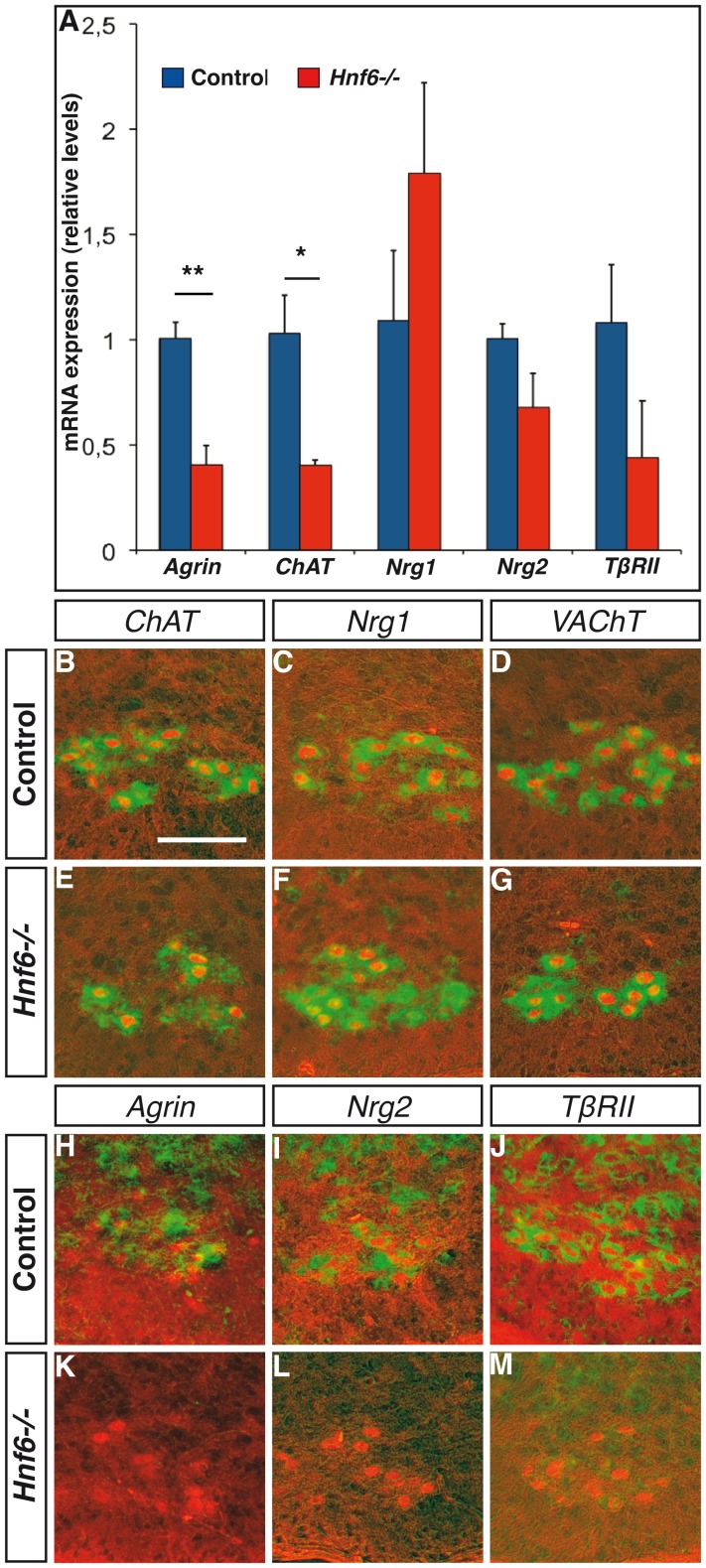
HNF-6 regulates in motor neurons the expression of several actors involved in NMJ formation. ***A*** **,** mRNA expression levels of *agrin*, *ChAT*, *neuregulin-1*, *neuregulin-2*, *TGF-ß receptor II* and *VAChT* in the spinal cord of control (blue) or *Hnf6−/−* (red) newborns at P0 (n = 3). Global expression levels of *agrin* and of *ChAT* are significantly reduced in the absence of HNF-6. ***B–M***, *In situ* hybridization (green) for *ChAT* (B,E), *neuregulin-1* (C,F), *VAChT* (D,G), *agrin* (H,K), *neuregulin-2* (I,L) or *TGF-ß receptor II* (J,M) combined to immunofluorescence detection of the motor neuron marker Isl1/2 (red) on sections in the lumbar spinal cord of control (B–D,H–J) or *Hnf6−/−* (E–G,K–M) P0 mice. Expression of *agrin*, *neuregulin-2* and *TGF-ß receptor II* is severely downregulated in the absence of HNF-6. ChAT: Choline acetyltransferase; Nrg-1: neuregulin-1; Nrg-2: neuregulin-2; TßRII: TGF-ß receptor II; VAChT: vesicular acetylcholine transporter. Student’s t-test; * = p<0.05, ** = p<0.01. Scale bar = 100 µm.

As HNF-6 is present at birth in spinal MN, we hypothesized that it could regulate the expression of some of the MN actors involved in NMJ formation. Global expression of *agrin* and of *choline acetyltransferase (ChAT)* significantly decreased in *Hnf6−/−* spinal cord at P0, while expression of *neuregulin-2 (nrg-2)* and of *TßRII* trended to decrease (p = 0.13 and 0.17, respectively)(Fig. 5A). To determine whether these perturbations specifically affected MN, *in situ* hybridization for these factors was combined to immunofluorescence for Isl1/2, which enabled to identify spinal MN, on lumbar spinal cord sections of P0 newborns. *Agrin*, *nrg-2* and *TßRII* expression was downregulated in *Hnf6−/−* MN and in surrounding ventral interneurons (Fig. 5B–M). These data indicate that HNF-6 stimulates in MN the expression of several factors involved in NMJ formation.

**Figure 6:**
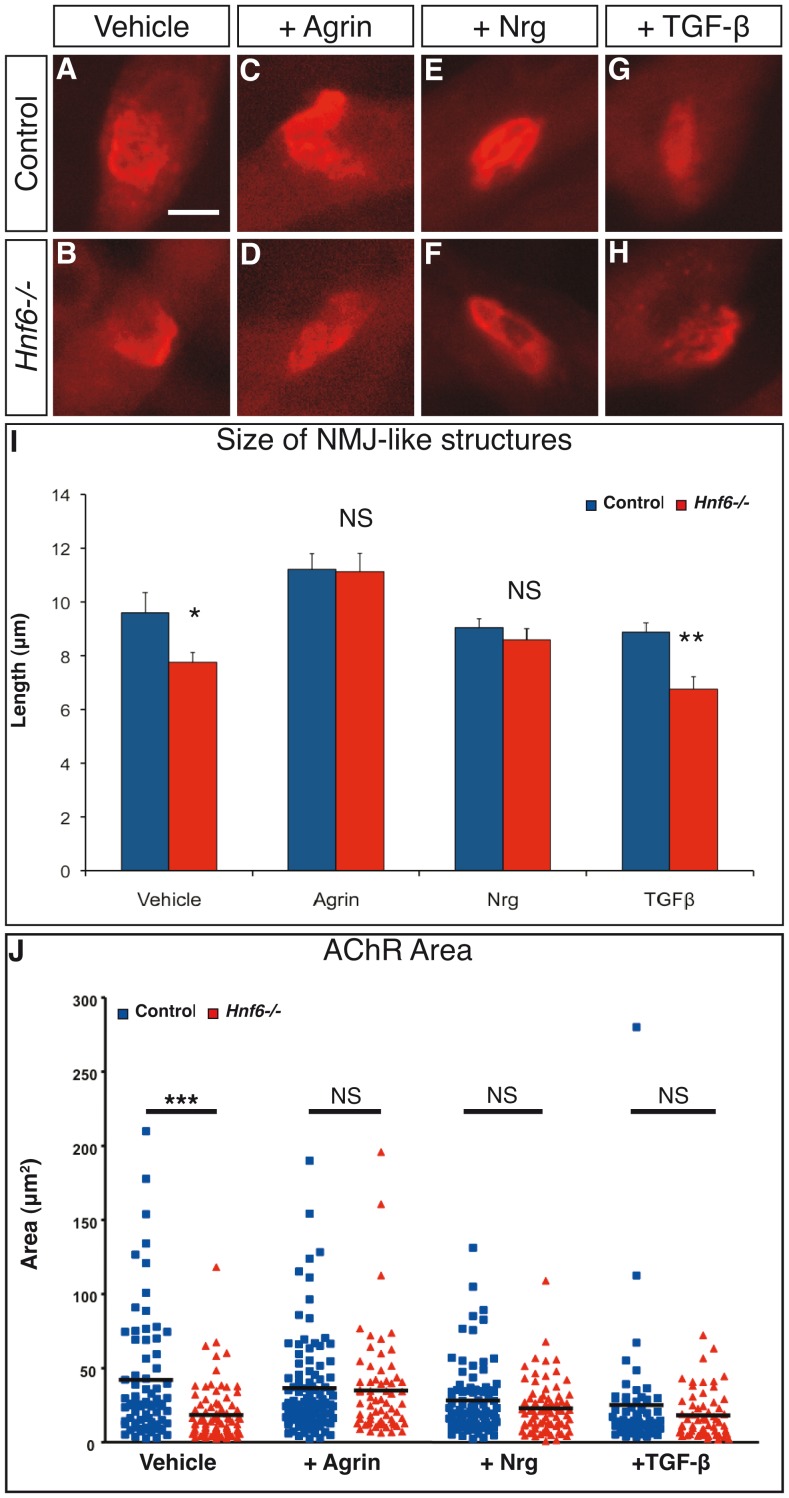
Depletion of agrin and of neuregulin contributes to defective NMJ formation in *Hnf6* mutant mice. ***A–H***, NMJ-like structures obtained *in vitro* by co-culture of control myotubes with control (A,C,E,G) or *Hnf6−/−* (B,D,F,H) embryonic spinal cord slices in medium supplemented with vehicle only (A,B) or with recombinant agrin (C,D), neuregulin (E,F) or TGFß (G,H). ***I*** **,** ***J,*** Quantification of the length (I) and of the area (J) of the NMJ-like structures obtained with control (blue) or *Hnf6−/−* (red) spinal cord slices. The size and area deficits observed with *Hnf6−/−* slices are fully rescued by agrin and by neuregulin, but not by TGFß (n = 3). Nrg-1: neuregulin-1. Dunett’s (I) and Newman-Keuls (J) multiple comparison tests; * = p<0.05, ** = p<0.01, *** = p<0.001. Scale bar = 10 µm.

To determine whether these perturbations contributed to the defective NMJ formation observed in *Hnf6−/−* mice, we relied on an *in vitro* co-culture system combining myotubes and embryonic spinal cord slices [48]. In this system, NMJ-like structures developed 2–3 weeks after plating slices of e12.5 control or *Hnf6−/−* spinal cord on differentiated control myotubes (Fig. 6). These structures are not detected when muscle cells are cultured alone, suggesting that their formation requires interactions of axon terminals with myotubes as observed *in vivo* for NMJ (data not shown). First, we assessed whether *in vitro* formation of these NMJ-like structures was affected by the absence of HNF-6 from the neuronal compartment. BTX labeling at the end of the culture period suggested that the formation of these structures was less efficient with *Hnf6−/−* than with control spinal cord slices (Fig. 6A,B). As the morphology of these structures was less well-established than that of their *in vivo* counterparts, their size and the surface covered by AChR were measured to evaluate their development [49]. The length and the surface of the NMJ-like structures after co-culture with *Hnf6−/−* spinal cord slices was significantly smaller than that obtained with control tissues (Fig. 6I,J). Thus, the growth of the NMJ-like structures obtained *in vitro* was decreased when HNF-6 was absent from the neuronal compartment. This was consistent with the hypothesis that HNF-6 acts in MN to control the development of the NMJ. It also validated our co-culture system to study the mechanisms that cause defective NMJ development in *Hnf6* mutant animals. To address this question, we assessed whether activation of the agrin, neuregulin or TGF-ß signaling pathway was able to rescue the growth deficit of NMJ-like structures observed with *Hnf6−/−* neuronal tissue. In co-cultures with control spinal cord slices, recombinant agrin, neuregulin or TGF-ß had no significant effect on the growth of NMJ-like structures (Fig. 6C–J). In co-cultures with *Hnf6−/−* tissues, recombinant agrin (Fig. 6C,D,I,J) and neuregulin (Fig. 6E,F,I,J) completely restored the growth defect of these structures (Fig. 6C–F,I,J), while TGF-ß was rather deleterious (Fig. 6G–J). These data indicate that activation of the agrin or neuregulin signaling pathways rescues the mutant phenotype in this *in vitro* co-culture system, and suggest that the downregulation of *agrin* and of *nrg-2* expression in *Hnf6−/−* newborn MN contributes to defective NMJ formation.

## Discussion

Onecut transcriptional activators control different aspects of CNS development including generation or maintenance of specific neuronal populations [24], [25], [26], [27], neuronal migration [24] and central projections of the trigeminal neurons [23]. Here, we present evidence that HNF-6 controls in MN part of the genetic program necessary for the development of hindlimb NMJ. Indeed, the formation of hindlimb junctions was altered and the expression of multiple factors described to participate in NMJ development, including *agrin*, *neuregulin*-2 and *TßRII*, was downregulated in the lumbar MN of *Hnf6* mutant newborns. Furthermore, recombinant agrin or neuregulin did rescue defective *in vitro* formation of NMJ-like structures due to the lack of HNF-6 from the neuronal compartment.

Only part of the hindlimb NMJ was altered in the *Hnf6* mutants, probably because HNF-6 is present in only part of the MN [28]. In particular, the NMJ of the soleus muscle were not affected. However, this could not be attributed to the absence of HNF-6 from the soleus MN pool in wild-type animals (data not shown). Similarly, the reason why forelimb NMJ were not affected remains unclear. Indeed, the distribution of HNF-6 in MN is similar at brachial or lumbar levels of the spinal cord [28]. Noteworthy, the distribution of other transcription factors including PEA3 [50], ER81 [51] and Hox factors [52] is different in brachial or lumbar limb-innervating MN, suggesting that the genetic programs that control forelimb and hindlimb innervation are not identical. Whether another factor compensates for the absence of HNF-6 in brachial MN remains to be determined. As the NMJ of the soleus were normal in *Hnf6−/−* mice, the hypertrophy of the soleus observed in these animals is probably secondary to the hypotrophy of the TA and gastrocnemius muscles.

Downregulation of *agrin*, *neuregulin*-2 and *TßRII* in *Hnf6−/−* MN suggests that these genes are, either directly or indirectly, regulated by HNF-6. Although RT-qPCR experiments showed a significant decrease in the expression levels of *agrin* and only a trend to the reduction for *neuregulin-2* and *TßRII*, *in situ* hybridization experiments indicated a strong reduction of these three factors in *Hnf6−/−* MN as well as in surrounding ventral interneurons, consistent with the expression of *Hnf6* in these neuronal populations [28]. However, *in situ* hybridization is only a semi-quantitative method with a relatively high detection threshold. Therefore, it is likely that the expression levels of *agrin*, *neuregulin-2* and *TßRII* are downregulated below this threshold in the *Hnf6−/−* MN, as indicated by the RT-qPCR experiment, but are not completely abrogated as suggested by the *in situ* hybridization data. This is consistent with the requirement for agrin in NMJ formation [5]. Although regulation of *agrin* or *neuregulin* genes by HNF-6 has not been described yet, *TßRII* is negatively regulated by HNF-6 in liver, probably indirectly [17], [39]. This regulation, along with that of TGF-ß antagonists, enables Onecut factors to maintain low TGF-ß signaling in the liver parenchyma and thereby creates an environment permissive for hepatocyte differentiation [17]. Our observations suggest that HNF-6 acts oppositely in MN and rather supports activation of the TGF-ß pathway.

In *Drosophila*, numerous studies have shown that TGF-ß signaling in MN regulates NMJ development. Mutations in genes that encode components of this pathway lead to presynaptic defects resulting in reduced numbers of NMJ, disrupted T-bars and impaired neurotransmitter release [53], [54], [55], [56]. Recently, it has been shown in *Xenopus* that TGFß-1 acts as a Schwann cell-derived signal that promotes NMJ formation, probably by stimulating *agrin* expression in MN [9]. Therefore, support of *TßRII* expression by HNF-6 may contribute to maintain MN responsive to this signal, and thereby to indirectly stimulate agrin production.

Agrin plays a pivotal role in AChR clustering and NMJ maintenance. Lack of agrin results in activity-dependant dispersal of AChR clusters, impaired postsynaptic differentiation and progressive loss of NMJ [57], [58]. Therefore, downregulation of *agrin* expression in *Hnf6* mutant mice may contribute to NMJ fragmentation. However, absence of severe NMJ depletion and of muscle denervation in these mutant animals suggests that *agrin* expression levels were only partly reduced in *Hnf6−/−* MN. Furthermore, agrin is also produced by the muscles and by terminal Schwann cells [59], which may partly compensate for the reduction in agrin signaling from MN in *Hnf6* mutant animals. Nevertheless, the growth defect of the NMJ-like structures observed *in vitro* with *Hnf6−/−* spinal cord slices was completely rescued by recombinant agrin, suggesting that decreased *agrin* levels contribute to the NMJ abnormalities in *Hnf6* mutant animals.

Expression level of *nrg-2* was also decreased in *Hnf6−/−* MN, and activation of neuregulin signaling rescued the mutant phenotype in co-culture experiments. *Nrg2−/−* mice show early growth retardation and reduced reproductive capacity [60], but NMJ were not examined in these mutants. However, as these animals are viable, their NMJ are probably functional. In contrast, *Nrg1+/−* mice showed a reduction in the density of AChR at the post-synaptic membrane [61]. Similarly, α-dystrobrevin-dependent AChR anchoring was severely altered in mice carrying inactivation of the Nrg receptors ErbB2 and ErbB4 in skeletal muscles, resulting in progressive NMJ wasting [6]. These data suggest that Nrg-1 and Nrg-2 may provide redundant signals that use the same intracellular signaling pathway to stabilize the post-synaptic apparatus. Therefore, downregulation of *Nrg-2* in *Hnf6−/−* mice may contribute to morphological alterations of adult NMJ but is unlikely to account for the early maturation defects of these junctions. Taken together, these data suggest that combined reduction in agrin, in neuregulin and in TGF-ß signaling causes the NMJ defects observed in the absence of HNF-6. This indicates that HNF-6 may act in lumbar MN to coordinate the genetic program that participates in hindlimb NMJ formation and maintenance.

Abnormal distribution of synaptophysin was observed in *Hnf6−/−* MN. Synaptophysin is one of the major integral membrane proteins of the small (30–50 nm diameter) electron-translucent transmitter-containing vesicles in neurons and accounts for ±10% of total synaptic vesicle proteins. The exact function of synaptophysin remains unclear. Mice lacking synaptophysin are healthy and show no apparent defect, suggesting that their NMJ are intact and functional although their organization was not studied [62], [63], [64], [65], [66]. In the absence of HNF-6, synaptophysin at the motor nerve terminals was either fragmented, reduced or absent, although the levels of *synaptophysin* mRNA in the spinal cord were similar in control and in *Hnf6−/−* newborn mice (data not shown). Abnormal synaptophysin distribution was not due to defective localization of the synaptic vesicles at the nerve terminals because other synaptic vesicle proteins, including VAChT and SV2, were properly distributed and a normal amount of synaptic vesicles was observed beneath the presynaptic membrane using electron microscopy. Therefore, abnormal synaptophysin distribution in *Hnf6−/−* mice seems to result from deficient translation or from defective targeting of synaptophysin toward the synaptic vesicles. How HNF-6 may contribute to these processes remains to be investigated.

Finally, the absence of HNF-6 caused abnormal locomotion and positioning of the hindlimbs. Some of the observed defects, including reduced muscle strength and posture defects, probably partly result from abnormal NMJ formation. However, other neuronal populations of the CNS are defective in *Hnf6−/−* animals [24], [25] (spinal interneurons in this study; data not shown) and likely contribute to the locomotion defects.

In conclusion, we have shown that the transcription factor HNF-6 coordinates in lumbar MN a genetic programs involved in hindlimb NMJ formation. Perturbations of this program caused defective formation of the NMJ that resulted in a reduction in the hindlimb muscle strength. Why this defect is restricted to hindlimb muscles, how HNF-6 controls in MN the expression of regulators of NMJ development and whether HNF-6 additionally modulates the expression of other regulators of NMJ formation remain to be investigated.

## Supporting Information

Figure S1 **HNF-6 is expressed in MN but not expressed in muscle.** ***A,B,*** Expression levels of *Hnf6* in liver (positive control), spinal cord or hindlimb muscles of P0 control animals (n = 3) evaluated on gel (A) and by quantitative real-time PCR (B). Global expression level of *Hnf6* is higher in liver than in the spinal cord. However, *Hnf6* expression is undetectable in hindlimb muscles. **C** ***–E*** **,** Detection of HNF-6 in e18.5 liver (positive control, C) or lumbar spinal cord (D) and in P0 hindlimb muscle (E) of control animals. HNF-6 is present in the liver and in the spinal cord. In contrast, it is not detected in the hindlimb muscles. Student’s t-test; * = p<0.05, ** = p<0.01. Scale bar = 100 µm.
(TIF)

Figure S2 **Spinal motor neurons are not affected by the absence of HNF-6.** ***A–D,*** Transverse sections in the lumbar spinal cord of e18.5 control (A,C) or *Hnf6−/−* (B,D) embryos labeled for the motor neuron markers Hb9 (A,B) or Isl1/2 (C,D). MN are present in the ventral horn of control (A,B) and of *Hnf6−/−* (C,D) mice. ***E,*** Quantification of motor neurons in hemisections in the lumbar spinal cord of control (blue) or *Hnf6−/−* (red) mice at e18.5. The amount of motor neurons was similar in control and in *Hnf6−/−* mice (n = 3). ***F,G*** **,** Transverse sections in the lumbar spinal cord of adult control (F) or *Hnf6−/−* (G) mice stained with cresyl violet. The dashed lines delineate the border between the gray and the white matter. MN (arrows) are present in the ventral horn of control and of *Hnf6−/−* mice. ***H,I,*** VAChT is detected in these cells, both in control (H) and in *Hnf6−/−* (I) mice, indicating that these are cholinergic neurons, as expected. ***J,K,*** Fluorogold was injected i.p. in control (J) or *Hnf6−/−* (K) mice. In both cases, 5 days after injection, the cell bodies of MN contain Fluorogold, indicating that these neurons project toward the periphery. ***L***, The amount of motor neurons was similar in control and in *Hnf6−/−* mice (n = 5). ***M,*** Table showing the weight of P70 control or *Hnf6−/−* mice. At that stage, the weight of *Hnf6−/−* mice is similar to that of control animals. VAChT: vesicular acetylcholine transporter. Scale bars = 100 µm.
(TIF)

Figure S3 **NMJ are altered in** ***Hnf6−/−*** **tibialis anterior, gastrocnemius and EDL muscles, but not in** ***Hnf6−/−*** **soleus muscle.** ***A–H’*** **,** Labeling of acetylcholine receptors by α-bungarotoxin (red) and immunofluorescence detection of synaptophysin (green) and of neurofilaments (blue) on tibialis anterior (A–B’), gastrocnemius (C–D’), EDL (E–F’) and soleus (G–H’) muscles of control (A–G’) or *Hnf6−/−* (B–H’) mice. ***A–G’*** **,** In control mice, NMJ display the expected “pretzel-like” shape and show perfect apposition of the synaptophysin labeling to the motor endplate. ***B–H’,*** In *Hnf6−/−* mice, junctions show disorganized topology and defective localization of synaptophysin (arrowhead) in tibialis anterior, gastrocnemius and EDL muscle. In contrast, the apposition of nerve terminals to the motor endplates and the endplate morphology are normal in *Hnf6−/−* soleus muscle. BTX: α-bungarotoxin; SYN: synaptophysin. PanNF: Pan Neurofilament; EDL: extensor digitorium longus. Scale bar = 5 µm.
(TIF)

Figure S4 **HNF-6 is not required for the formation of forelimb and diaphragm NMJ.** ***A–D’*** **,** Labeling of acetylcholine receptors by α-bungarotoxin (red) and immunofluorescence detection of synaptophysin (green) on forelimb muscle sections (A–B’) or on diaphragm (C–D’) of control (A–A’,C–C’) or *Hnf6−/−* (B–B’,D–D’) mice at postnatal days 14. The apposition of the nerve terminals to the motor endplates and the endplate maturation are normal in forelimb and in diaphragm NMJ of *Hnf6−/−* mice. BTX: α-bungarotoxin; SYN: synaptophysin. Scale bar = 5 µm.
(TIF)
