# Supplementary figures and images for: The Onecut Transcription Factor HNF-6 Regulates in Motor Neurons the Formation of the Neuromuscular Junctions
Source: PLoS One. 2012 Dec 5;7(12):e50509. doi: 10.1371/journal.pone.0050509 (PMC3515622; doi:10.1371/journal.pone.0050509)

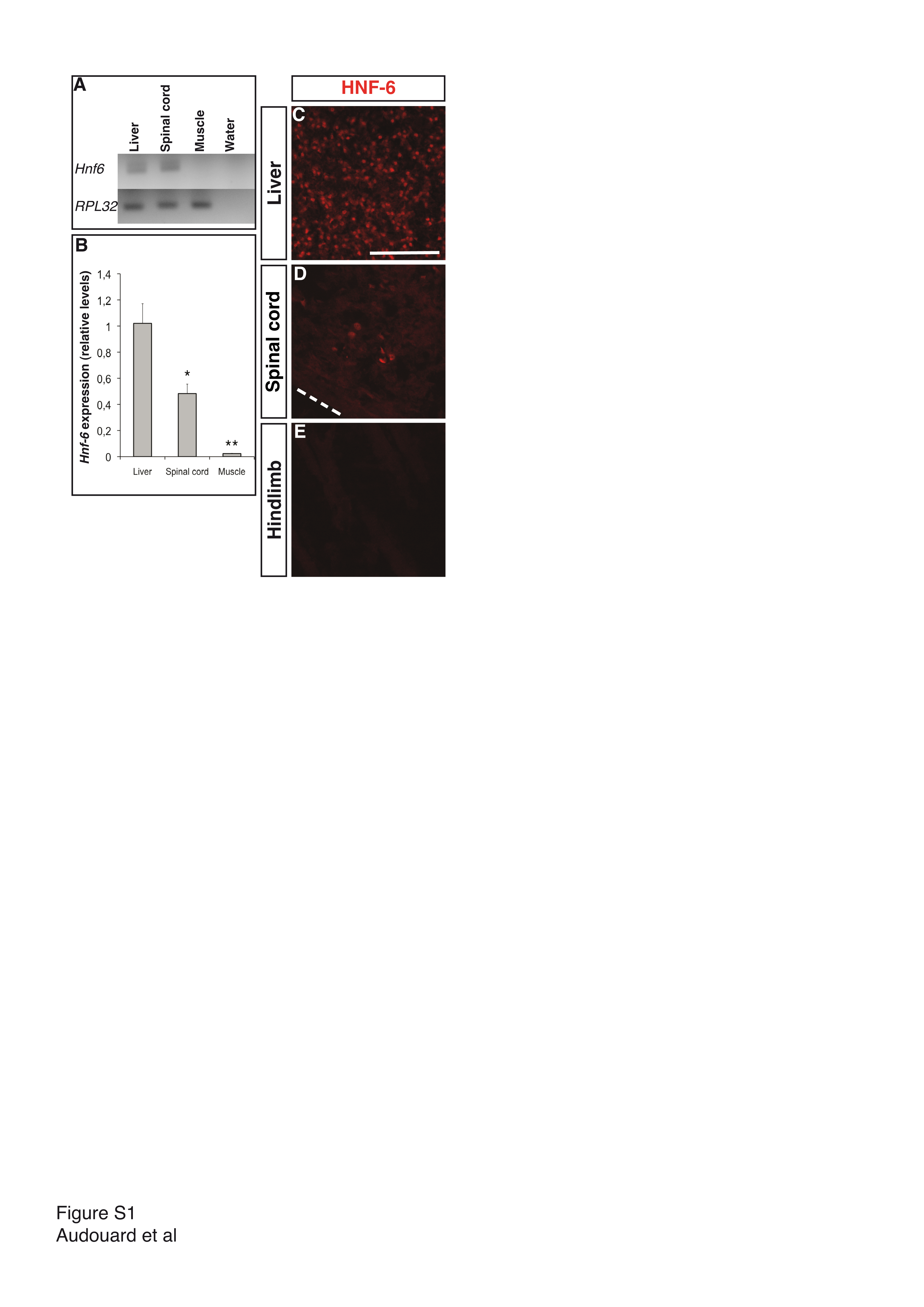

Supplement: Figure S1 — HNF-6 is expressed in MN but not expressed in muscle. A,B, Expression levels of Hnf6 in liver (positive control), spinal cord or hindlimb muscles of P0 control animals (n = 3) evaluated on gel (A) and by quantitative real-time PCR (B). Global expression level of Hnf6 is higher in liver than in the spinal cord. However, Hnf6 expression is undetectable in hindlimb muscles. C –E , Detection of HNF-6 in e18.5 liver (positive control, C) or lumbar spinal cord (D) and in P0 hindlimb muscle (E) of control animals. HNF-6 is present in the liver and in the spinal cord. In contrast, it is not detected in the hindlimb muscles. Student’s t-test; * = p<0.05, ** = p<0.01. Scale bar = 100 µm. (TIF) [file pone.0050509.s001.tif]

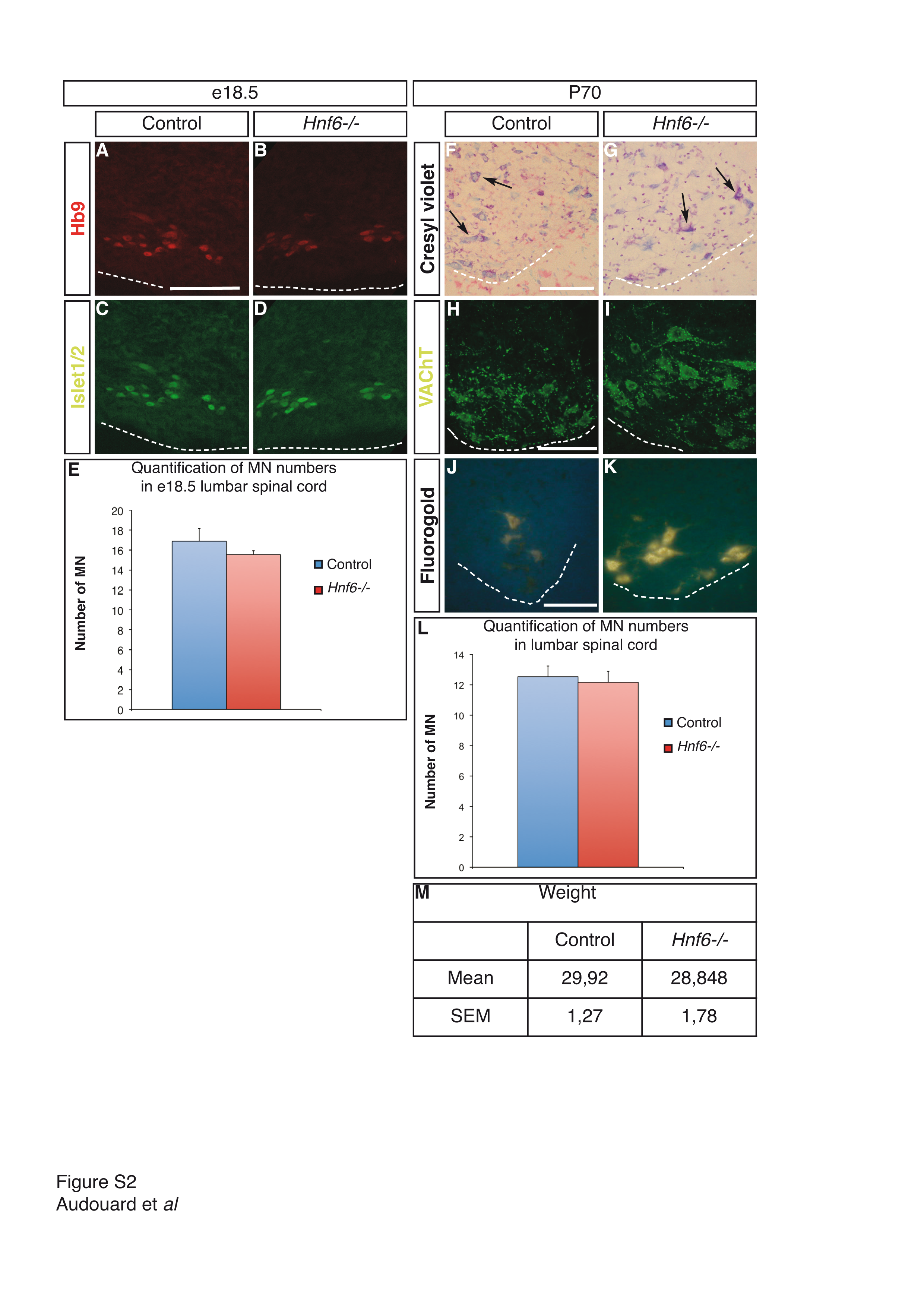

Supplement: Figure S2 — Spinal motor neurons are not affected by the absence of HNF-6. A–D, Transverse sections in the lumbar spinal cord of e18.5 control (A,C) or Hnf6−/− (B,D) embryos labeled for the motor neuron markers Hb9 (A,B) or Isl1/2 (C,D). MN are present in the ventral horn of control (A,B) and of Hnf6−/− (C,D) mice. E, Quantification of motor neurons in hemisections in the lumbar spinal cord of control (blue) or Hnf6−/− (red) mice at e18.5. The amount of motor neurons was similar in control and in Hnf6−/− mice (n = 3). F,G , Transverse sections in the lumbar spinal cord of adult control (F) or Hnf6−/− (G) mice stained with cresyl violet. The dashed lines delineate the border between the gray and the white matter. MN (arrows) are present in the ventral horn of control and of Hnf6−/− mice. H,I, VAChT is detected in these cells, both in control (H) and in Hnf6−/− (I) mice, indicating that these are cholinergic neurons, as expected. J,K, Fluorogold was injected i.p. in control (J) or Hnf6−/− (K) mice. In both cases, 5 days after injection, the cell bodies of MN contain Fluorogold, indicating that these neurons project toward the periphery. L, The amount of motor neurons was similar in control and in Hnf6−/− mice (n = 5). M, Table showing the weight of P70 control or Hnf6−/− mice. At that stage, the weight of Hnf6−/− mice is similar to that of control animals. VAChT: vesicular acetylcholine transporter. Scale bars = 100 µm. (TIF) [file pone.0050509.s002.tif]

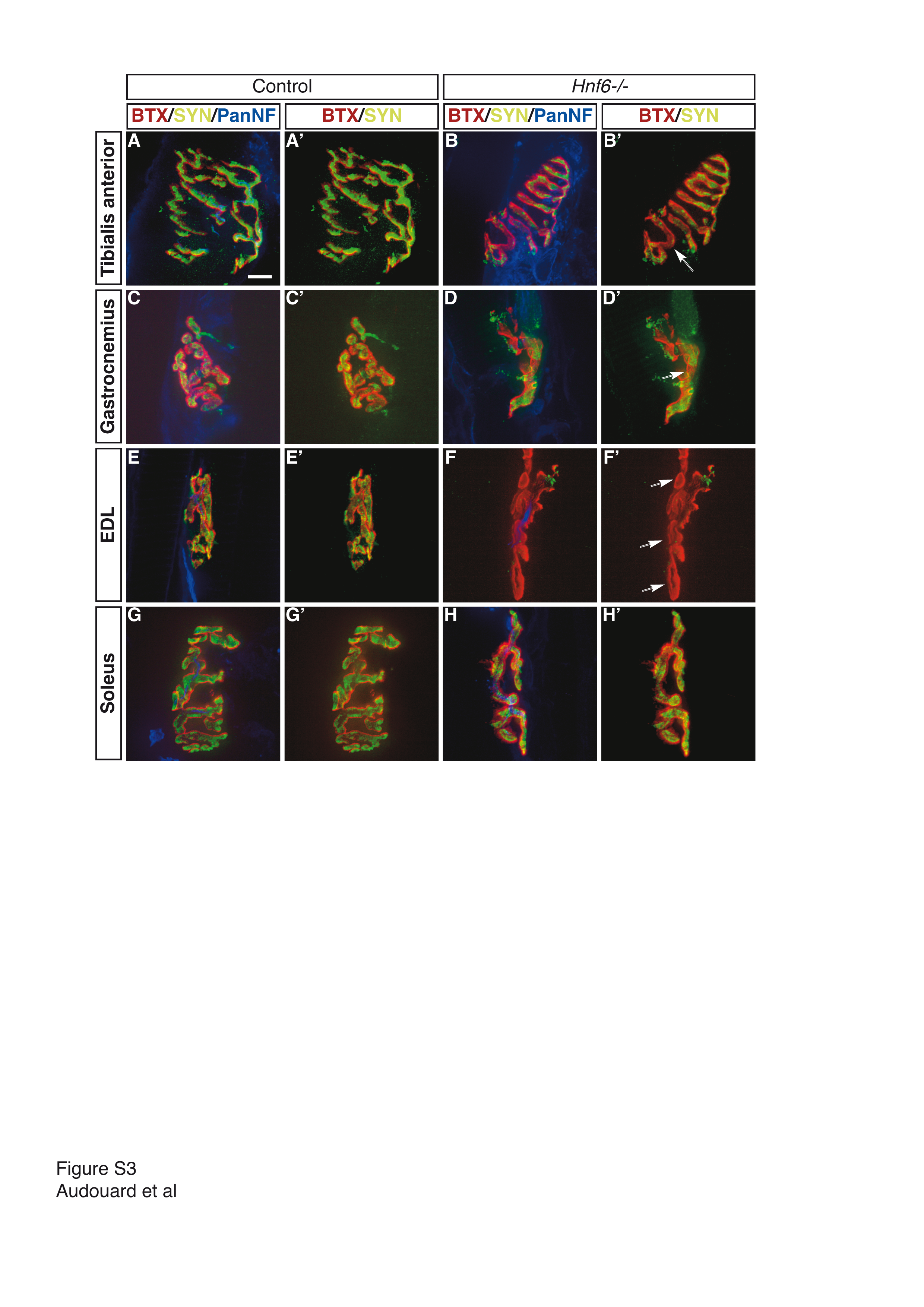

Supplement: Figure S3 — NMJ are altered in Hnf6−/− tibialis anterior, gastrocnemius and EDL muscles, but not in Hnf6−/− soleus muscle. A–H’ , Labeling of acetylcholine receptors by α-bungarotoxin (red) and immunofluorescence detection of synaptophysin (green) and of neurofilaments (blue) on tibialis anterior (A–B’), gastrocnemius (C–D’), EDL (E–F’) and soleus (G–H’) muscles of control (A–G’) or Hnf6−/− (B–H’) mice. A–G’ , In control mice, NMJ display the expected “pretzel-like” shape and show perfect apposition of the synaptophysin labeling to the motor endplate. B–H’, In Hnf6−/− mice, junctions show disorganized topology and defective localization of synaptophysin (arrowhead) in tibialis anterior, gastrocnemius and EDL muscle. In contrast, the apposition of nerve terminals to the motor endplates and the endplate morphology are normal in Hnf6−/− soleus muscle. BTX: α-bungarotoxin; SYN: synaptophysin. PanNF: Pan Neurofilament; EDL: extensor digitorium longus. Scale bar = 5 µm. (TIF) [file pone.0050509.s003.tif]

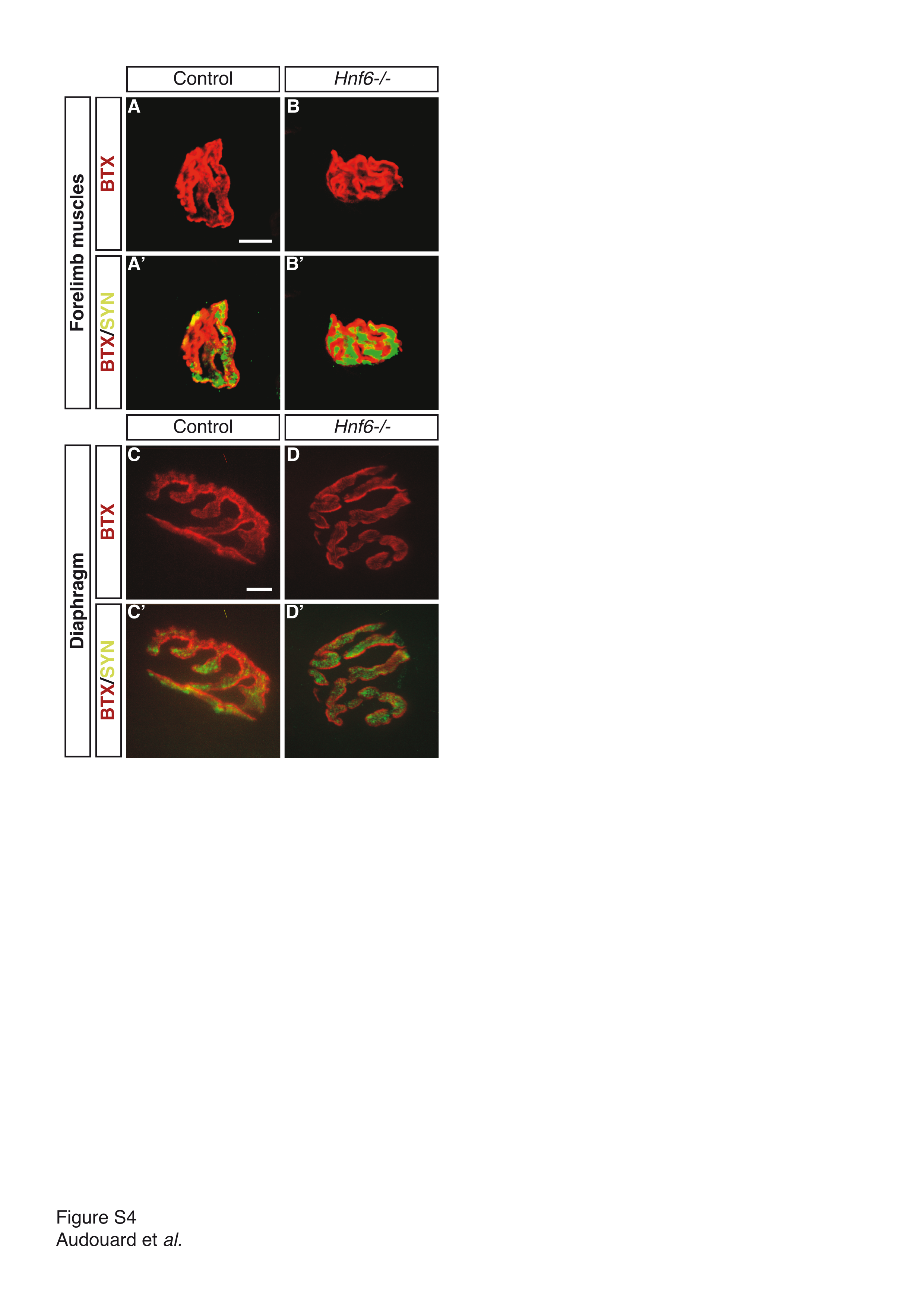

Supplement: Figure S4 — HNF-6 is not required for the formation of forelimb and diaphragm NMJ. A–D’ , Labeling of acetylcholine receptors by α-bungarotoxin (red) and immunofluorescence detection of synaptophysin (green) on forelimb muscle sections (A–B’) or on diaphragm (C–D’) of control (A–A’,C–C’) or Hnf6−/− (B–B’,D–D’) mice at postnatal days 14. The apposition of the nerve terminals to the motor endplates and the endplate maturation are normal in forelimb and in diaphragm NMJ of Hnf6−/− mice. BTX: α-bungarotoxin; SYN: synaptophysin. Scale bar = 5 µm. (TIF) [file pone.0050509.s004.tif]
